# The association between self-reported sleep problems, infection, and antibiotic use in patients in general practice

**DOI:** 10.3389/fpsyt.2023.1033034

**Published:** 2023-03-02

**Authors:** Ingeborg Forthun, Knut Eirik Ringheim Eliassen, Knut Erik Emberland, Bjørn Bjorvatn

**Affiliations:** ^1^Department of Global Public Health and Primary Care, University of Bergen, Bergen, Norway; ^2^Department of Disease Burden, Norwegian Institute of Public Health, Bergen, Norway; ^3^Norwegian Competence Center for Sleep Disorders, Haukeland University Hospital, Bergen, Norway

**Keywords:** sleep initiation and maintenance disorders, infection, antibiotics, primary care, insomnia

## Abstract

**Objectives:**

There is emerging evidence that sleep problems and short sleep duration increase the risk of infection. We aimed to assess whether chronic insomnia disorder, chronic sleep problems, sleep duration and circadian preference based on self-report were associated with risk of infections and antibiotic use among patients visiting their general practitioner (GP).

**Methods:**

We conducted a cross-sectional study of 1,848 unselected patients in Norway visiting their GP during 2020.The patients completed a one-page questionnaire while waiting for the consultation, that included the validated Bergen Insomnia Scale (BIS), questions on self-assessed sleep problem, sleep duration and circadian preference and whether they have had any infections or used antibiotics in the last 3 months. Relative risks (RR) were estimated using modified Poisson regression models.

**Results:**

The risk of infection was 27% (95% CI RR 1.11–1.46) and 44% higher (95% CI 1.12–1.84) in patients sleeping < 6 h and >9 h, respectively, compared to those sleeping 7–8 h. The risk was also increased in patients with chronic insomnia disorder or a chronic sleep problem. For antibiotic use, the risk was higher for patients sleeping < 6 h, and for those with chronic insomnia disorder or a chronic sleep problem.

**Conclusions:**

Among patients visiting their GP, short sleep duration, chronic insomnia and chronic sleep problem based on self-report were associated with higher prevalence of infection and antibiotic use. These findings support the notion of a strong association between sleep and infection.

## 1. Introduction

Sleep is an important determinant of health and wellbeing (1). Insomnia is the most common sleep problem with a prevalence of 10–20% in the general population (2–4). In Norway, among patients in general practice, the prevalence of insomnia has been found to be as high as 54% (5, 6). The prevalence varies according to factors such as sex, age, socioeconomic status and circadian preference—evening types are reported to be more susceptible (2, 7). There is emerging evidence, both from controlled laboratory and epidemiological observational studies, that sleep disturbances and short sleep duration increase the risk of infection (8–17). The underlying mechanisms behind this association are unclear and is further complicated by the bidirectional relationship between the two (1). Several studies have found an increase in inflammatory markers associated with sleep disturbances, but overall, the results have varied (1, 18). Infections are a common reason for visits to general practice (19, 20), where also a large percentage of antibiotics are prescribed (21). Sleep disturbances are in many instances treatable (3). A causal effect of sleep disturbances on the immune system would therefore imply an opportunity to reduce the risk of infection and the use of antibiotics (22).

Most of the previous observational studies on sleep and risk of infection have been conducted in samples of the adult general population. None have included circadian preference or antibiotic use, and few have used validated instruments to assess sleep disturbances. Thus, in the present study we aimed to assess whether sleep duration, chronic insomnia disorder [based on the Diagnostic and Statistical Manual for Mental disorders (DSM)-version-5], chronic sleep problems and circadian preference based on self-report were associated with risk of infections and antibiotic use among patients visiting their general practitioner (GP).

## 2. Material and methods

### 2.1. Participants

The study was based on data collected by last year medical students at the University of Bergen who in the spring and fall semester of 2020 were deployed in general practices in Western Norway for 6 weeks. In Norway, the GPs are organized in a list-based system in which all citizens are entitled to a general practitioner (GP). While deployed, the students were asked to collect questionnaire data from 20 consecutive and unselected patients. Patients were recruited in the waiting room of the GP regardless of their reason for the appointment and asked to answer a one-page questionnaire with questions on background factors, infections, and sleep. Of 153 students, 114 collected data for the study.

### 2.2. Measures

#### 2.2.1. Sleep-related variables

Information on sleep duration was based on the question “Approximately how long do you sleep per day?” with the answer categories “ < 6 h”, “6–7 h”, “7–8 h” (reference category), “8–9 h” and “>9 h”. Circadian preference (morningness-eveningness) (“Are you a morning (lark) or evening (owl) type?”) was self-reported on a 5-point scale (“definitively a morning type”, “more a morning than an evening type”, “neither a morning nor an evening type”, “more an evening than a morning type” or “definitely an evening type”). To get a larger sample within each group, we created a new variable with three categories with “morning type (lark)” (first two categories), “neither a morning nor an evening type” (middle category) (reference category), and “night type (owl)” (last two categories). Chronic insomnia disorder was measured using the validated Bergen Insomnia Scale (BIS) (23) that was developed according to the Diagnostic and Statistical Manual of Mental Disorders, 4th edition (DSM-IV) (24). In the present study the Bergen Insomnia Scale was adapted according to the updated DSM-5 diagnostic criteria (25). The updated scale includes six items that are scored along an eight-point scale indicating the number of days per week during the past 3 months for which a specific insomnia symptom is experienced (0–7 days). Those who reported 3 days or more per week on at least one of the first three items (sleep onset latency >30 min, wake after sleep onset >30 min, early morning awakening >30 min) and 3 days or more per week on at least one of the two latter items (problematic tiredness/sleepiness, dissatisfaction with sleep) were defined as having chronic insomnia disorder (25). Cronbach's α for BIS was 0.88 in the present sample. The participants were also asked for how long they have had a sleep problem with four answer categories (“do not have sleep problems”, “ < 3 months”, “3 months to a year” or “more than a year”). A variable for chronic sleep problem was generated in which those who reported to have had a sleep problem for 3 months or more on this question were coded “yes”.

#### 2.2.2. Infections and antibiotics

Information on infections during the last 3 months was collected using a table in which the respondents were asked to indicate number of times (0, 1, 2, 3, >3 times) they have had the following infections: common cold, throat, otitis or sinusitis, pneumonia/bronchitis, eye infection, gastrointestinal infection with vomit or diarrhea, urinary tract infection, skin infection or any other infection. The main outcome was any type of infection (“yes”, “no”, including all types of infections). Respiratory (RTI) (common cold, throat, otitis or sinusitis and pneumonia/bronchitis), gastrointestinal (GI) and urinary tract infections (UTI) were looked at specifically as they constituted the three largest infection groups (eye infection (4.3%) and skin infections (7.0 %) were not looked at separately as there were so few who reported these). Information on antibiotics in the last 3 months (“yes”, “no”) was based on a question asking whether they had used antibiotics in the last 3 months (“no”, “yes, one prescription”, “yes, two prescriptions”, “yes, three or more prescriptions”).

### 2.3. Ethics

The study was approved by The Regional Committee for Medical and Health Research Ethics in Western Norway (REK) (ref. 61165).

### 2.4. Statistical analysis

Modified Poisson regression models (26) were used to estimate relative risks (RR) with 95% confidence intervals (CI) for the association between the sleep related variables and infection. The analyses were conducted in R statistical package (R Foundation for Statistical Computing) and Stata/SE version 17. In the adjusted analyses we adjusted for sex (“male”, “female”), age (continuous variable, included both age and age squared to account for non-linearity in associations), educational level (“primary or lower secondary education”, “upper secondary education”, “vocational school”, “higher education”), children living at home (“yes”, “no”) and season of data collection [“spring” (February-May), “fall” (September–December)].

## 3. Results

A total of 2201 questionnaires were collected, of which 1,875 were answered (response rate 85.2%). The analytic sample included 1848 patients after excluding those who were under 18 years. Of the total sample, 60.6% were female, the mean age was 52 years (standard deviation (SD) 18), 38.0% had higher education and 33.3% children living at home (Table 1). A total of 21.0% reported a sleep duration of < 6 h, and 2.0% >9 h. Chronic insomnia disorder based on the DSM-5 criteria was present in 48.3% of the participants, while 46.9% fulfilled the criteria for chronic sleep problem. The prevalence of self-report of any type of infection, RTI, GI and UTI in the last 3 months were 53.9, 35.9, 11.0, and 9.8%, respectively, and 16.0% reported to have used antibiotics at least once during this period.

**Table 1:** Patient characteristics of 1,848 patients in Norway visiting their general practitioner during the spring and fall of 2020, total and for any type of infection and use of antibiotics.

**Characteristic**	**Total *n* = 1,848**	**Any infection^a^ *n* = 954**	**Use of antibiotics *n* = 296**
**Sex**, ***n*** **(col %)**			
Female	1,098 (60.6)	579 (61.8)	183 (63.8)
Male	714 (39.4)	358 (38.2)	104 (36.2)
Data missing	36	17	9
Age in years, mean (SD)	52 (18)	49 (18)	55 (18)
Data missing	69	35	18
**Season**, ***n*** **(col %)**			
Spring	887 (48.0)	501 (52.5)	136 (46.0)
Fall	961 (52.0)	453 (47.5)	160 (54.1)
**Education**, ***n*** **(col %)**			
Primary and lower secondary education	189 (11.1)	92 (10.4)	37 (14.1)
Upper secondary education	505 (29.6)	246 (27.9)	65 (24.8)
Vocational school	363 (21.3)	197 (22.3)	74 (28.2)
Higher education	648 (38.0)	348 (39.4)	86 (32.8)
Data missing	143	71	34
**Children living at home**, ***n*** **(col %)**			
Yes	550 (33.3)	313 (36.4)	79 (30.9)
No	1,100 (66.7)	547 (63.6)	177 (69.1)
Data missing	198	94	40
**Sleep duration**, ***n*** **(col %)**			
< 6 h	377 (21.0)	223 (23.8)	82 (28.3)
6–7 h	828 (46.1)	427 (45.5)	120 (41.4)
7–8 h	448 (24.9)	209 (22.3)	66 (22.8)
8–9 h	108 (6.0)	53 (5.7)	18 (6.2)
>9 h	35 (2.0)	26 (2.8)	4 (1.4)
Data missing	52	16	6
**Morning type (lark) or evening type (owl)**, ***n*** **(col %)**			
Morning type	747 (41.7)	362 (38.7)	128 (44.0)
Neither morning nor evening type	372 (20.8)	195 (20.9)	63 (21.7)
Evening type	672 (37.5)	378 (40.4)	100 (34.4)
Data missing	57	19	5
**Chronic insomnia disorder**, ***n*** **(col %)**			
Yes	829 (48.3)	468 (52.3)	157 (56.7)
No	887 (51.7)	427 (47.7)	120 (43.3)
Data missing	132	59	19
**Chronic sleep problem**^b^, ***n*** **(col %)**			
Yes	829 (46.9)	456 (49.5)	149 (53.4)
No	940 (53.1)	465 (50.5)	130 (46.6)
Data missing	79	33	17

a Include those who reported that they have had common cold, throat infection, lung infection, eye infection, gastrointestinal infection, urinary tract infection, skin infection or other infection.

b Sleep problem of ≥3 months.

**Figure 1:**
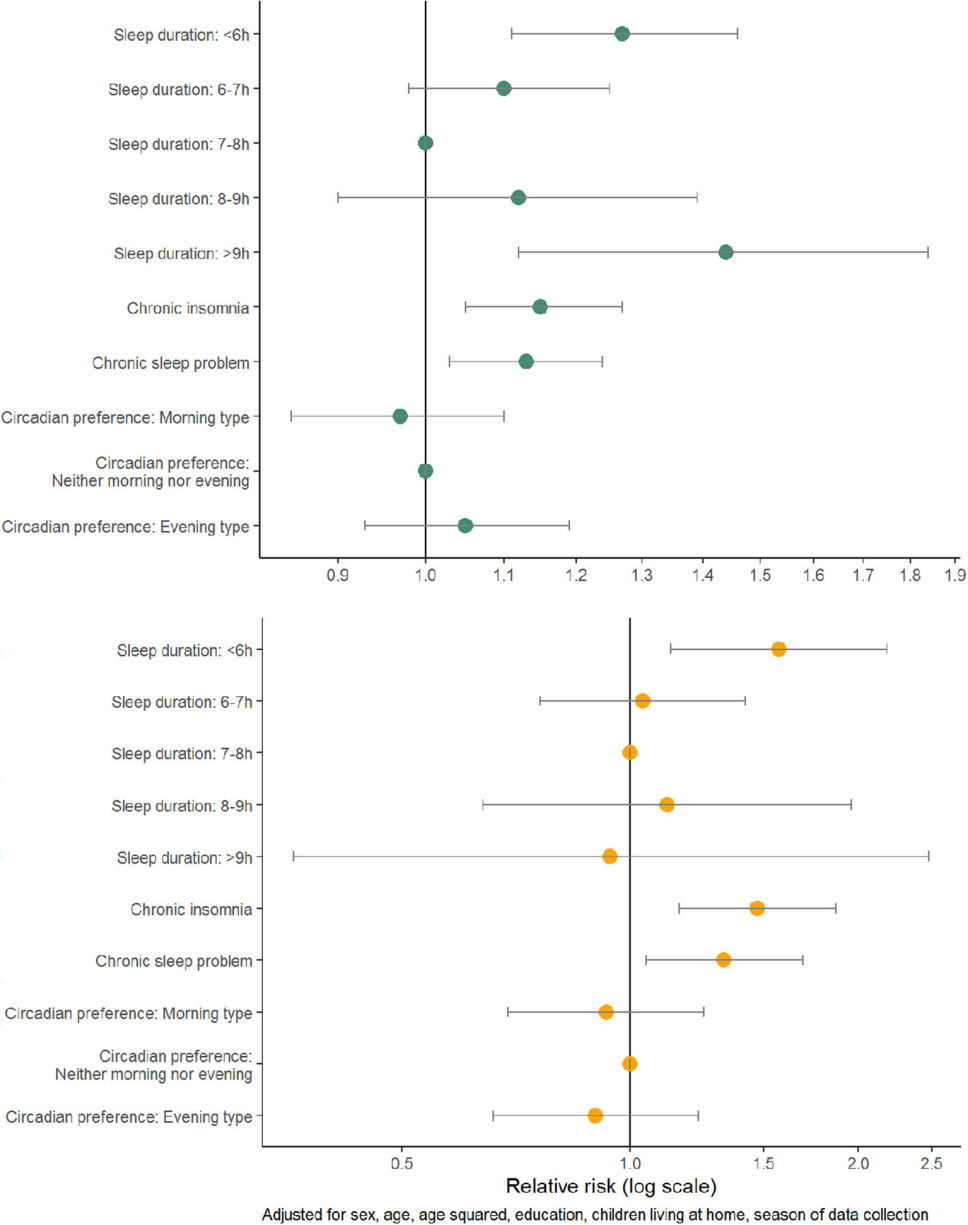
Adjusted relative risks with 95% confidence interval of any type of infection **(top panel)** and antibiotic use **(bottom panel)** among 1,848 patients visiting their GPs in the spring and fall of 2020.

The absolute risk of any type of infection and antibiotic use varied within most of the explanatory variables (Table 2). Patients who reported a sleep duration of < 6 h had an increased risk of infection (adjusted RR (aRR) 1.27, 1.11–1.46) and antibiotic use (aRR 1.57, 95% CI 1.13–2.18) compared to those with sleep duration of 7–8 h (Figure 1, Supplementary Table 1). The risk of infection was also increased in those with >9 h (aRR 1.44, 95% CI 1.12–1.84). Patients with chronic insomnia disorder and with a chronic sleep problem had an increased risk of infection (aRR 1.15, 95% CI 1.05–1.27, aRR 1.13, 95% CI 1.03–1.24) and antibiotic use (aRR 1.47, 95% CI 1.16–1.87, aRR 1.33, 95% CI 1.05–1.69).

**Table 2:** Absolute risk of infection (any type) and antibiotic use among 1,848 patients visiting their GPs in the spring and fall of 2020.

**Characteristics**	**Reporting infections, *n* (%)**	**Reporting use of antibiotics, *n* (%)**
**Sleep duration**		
< 6 h	223 (62.1)	82 (22.2)
6–7 h	427 (53.2)	120 (14.8)
7–8 h	209 (48.6)	66 (15.0)
8–9 h	53 (51.5)	18 (17.1)
>9 h	26 (74.3)	4 (11.8)
**Chronic insomnia disorder**		
Yes	468 (58.2)	157 (19.4)
No	427 (49.9)	120 (13.8)
**Chronic sleep problem**		
Yes	456 (57.3)	149 (18.4)
No	465 (51.3)	130 (14.1)
**Circadian preference**		
Morning type	362 (50.8)	128 (17.6)
Neither morning nor evening type	195 (53.9)	63 (17.2)
Evening type	378 (57.7)	100 (15.1)

In analyses of specific infections, there was some indication of an association between sleep duration and risk of RTI, but the association was not statistically significant (Table 3). For patients with a sleep duration of < 6 h or >9 h, and for those with a chronic sleep problem, the risk of GI was increased (Table 3). For UTI, the risk was higher for patients with chronic insomnia disorder.

**Table 3:** Absolute risk, crude and adjusted relative risk (RR) with 95% confidence interval (CI) for respiratory, gastrointestinal and urinary tract infection among 1,848 patients visiting their GPs in the spring and fall of 2020, statistically significant results are indicated in bold.

**Characteristics**	**Reporting infections, *n* (%)**	**Crude RR (95% CI)**	**Adjusted^a^ RR (95% CI)**
**Respiratory infections**			
**Sleep duration**			
< 6 h	139 (38.7)	1.16 (0.96–1.39)	1.16 (0.96–1.42)
6–7 h	295 (36.7)	1.10 (0.93–1.29)	1.13 (0.96–1.33)
7–8 h	144 (33.5)	1.00 (ref.)	1.00 (ref.)
8–9 h	32 (31.1)	0.93 (0.68–1.27)	1.02 (0.74–1.39)
>9 h	15 (42.9)	1.28 (0.85–1.92)	1.23 (0.80–1.89)
**Chronic insomnia disorder**			
Yes	308 (38.3)	1.12 (0.99–1.28)	1.07 (0.94–1.22)
No	292 (34.1)	1.00 (ref.)	1.00 (ref.)
**Chronic sleep problem**			
Yes	292 (36.7)	1.03 (0.90–1.16)	1.06 (0.93–1.20)
No	324 (35.8)	1.00 (ref.)	1.00 (ref.)
**Circadian preference**			
Morning type	222 (31.1)	0.87 (0.73–1.03)	0.95 (0.79–1.14)
Neither morning nor evening type	130 (35.9)	1.00 (ref.)	1.00 (ref.)
Evening type	271 (41.4)	1.15 (0.98–1.36)	1.10 (0.93–1.30)
**Gastrointestinal infection**			
**Sleep duration**			
< 6 h	62 (16.9)	**1.90 (1.30**–**2.76)**	**1.92 (1.29**–**2.87)**
6–7 h	75 (9.3)	1.04 (0.72–1.51)	1.05 (0.71–1.55)
7–8 h	39 (8.9)	1.00 (ref.)	1.00 (ref.)
8–9 h	12 (11.7)	1.31 (0.71–2.41)	1.51 (0.80–2.83)
>9 h	8 (22.9)	**2.57 (1.30**–**5.06)**	**2.33 (1.12**–**4.85)**
**Chronic insomnia disorder**			
Yes	108 (13.3)	**1.37 (1.05**–**1.79)**	1.30 (0.97–1.73)
No	84 (9.7)	1.00 (ref.)	1.00 (ref.)
**Chronic sleep problem**			
Yes	112 (13.8)	**1.49 (1.14**–**1.94)**	**1.59 (1.20**–**2.11)**
No	85 (9.3)	1.00 (ref.)	1.00 (ref.)
**Circadian preference**			
Morning type	64 (8.9)	0.72 (0.50–1.03)	0.82 (0.55–1.21)
Neither morning nor evening type	45 (12.3)	1.00 (ref.)	1.00 (ref.)
Evening type	84 (12.7)	1.03 (0.73–1.44)	1.10 (0.76–1.59)
**Urinary tract infection**			
**Sleep duration**			
< 6 h	49 (13.5)	1.31 (0.90–1.92)	1.17 (0.77–1.78)
6–7 h	70 (8.6)	0.84 (0.59–1.20)	0.74 (0.50–1.10)
7–8 h	45 (10.3)	1.00 (ref.)	1.00 (ref.)
8–9 h	6 (5.8)	0.57 (0.25–1.29)	0.52 (0.22–1.24)
>9 h	5 (14.7)	1.43 (0.61–3.36)	1.42 (0.61–3.31)
**Chronic insomnia disorder**			
Yes	94 (11.6)	**1.52 (1.13**–**2.06)**	**1.41 (1.01**–**1.95)**
No	66 (7.6)	1.00 (ref.)	1.00 (ref.)
**Chronic sleep problem**			
Yes	84 (10.5)	1.14 (0.86–1.52)	1.07 (0.78–1.48)
No	84 (9.2)	1.00 (ref.)	1.00 (ref.)
**Circadian preference**			
Morning type	76 (10.6)	1.20 (0.81–1.78)	1.54 (0.95–2.48)
Neither morning nor evening type	32 (8.8)	1.00 (ref.)	1.00 (ref.)
Evening type	63 (9.6)	1.09 (0.72–1.63)	1.34 (0.82–2.21)

a Adjusted for sex, age and age squared, education, children living at home, season of data collection.

## 4. Discussion

In this cohort of 1,848 unselected patients in general practice with self-reported data, we found that both short and long sleep duration, chronic insomnia disorder and having a chronic sleep problem were associated with increased risk of reporting any type of infection in the last 3 months. Short sleep duration, chronic insomnia disorder and chronic sleep problem were also associated with increased risk of antibiotic use. The risk of gastrointestinal infections was increased in patients who reported short (< 6 h) and long sleep (≥9 h), and for those who reported a chronic sleep problem. For urinary tract infections, the risk was increased in patients with chronic insomnia disorder.

### 4.1. Strengths and limitations

Strengths of the study include a high response rate (85.2%) and the fact that we included an unselected sample of patients visiting their GPs. Therefore, the results are likely generalizable to patients in general practice. A validated instrument—the Bergen Insomnia Scale (BIS)—was used to assess insomnia based on the DSM-5 criteria (validated based on DSM-IV) (23). The measure used to assess circadian preference has not been validated against other circadian measures but has been used in several previous publications (6, 7, 27).

We do not know why the patient visited their GP and our study did not include any clinical assessment of sleep problems, chronotype, nor infection. At the same time, we were able to assess clinically relevant measures using a questionnaire. Some of the results were imprecise with broad confidence intervals due to a small sample in some of the sub-groups of exposure-outcome combinations. In addition, our measure of RTI included very broad and crude categories of infections. There could be a problem with recall bias in which patients with a self-assessed sleep problem are more or less likely to recall episodes of infection or antibiotic use. If so, this could result in bias.

The data were collected during the 1st year of the COVID-19 pandemic. The pandemic may have made patients more aware of symptoms of respiratory infections. At the same time, it probably also resulted in a lower prevalence of common RTIs due to infection control measures. It is unlikely that this have affected the observed associations in the present study. However, an increase in virtual online consultations (28) may have affected the representatives of our sample. We found a higher proportion of older patients in the present study compared to a similar Norwegian study from 2014 with the same design and source population (5). This was less pronounced for the spring semester (February–May 2020) that also included data collected before the first COVID cases were detected in Norway. All adjusted analyses included semester as a covariate, and this had little effect on the estimates.

### 4.2. Comparison with existing literature

Most of the previous observational studies on the association between sleep parameters and risk of infection have looked at respiratory infections and have generally reported a higher risk in short sleepers or in those with a self-reported sleep problem (13–15, 17, 29). The lack of statistically significant associations for risk of RTI in the present study was therefore surprising. We have identified two previous studies within patient populations and one study with a sample from the general population including records from hospitals and primary care. In a Taiwanese registry-based study, including a random sample of 8,061 patients with an insomnia diagnosis and 16 112 without such a diagnosis, the risk of pneumonia was more than doubled in those with an insomnia diagnosis (13). In that study, the diagnoses of both pneumonia and insomnia were made by a clinician and not self-reported, hence it is likely that only the most severe cases were included. In a Japanese study including 39 524 adults without any serious medical conditions who underwent a medical health checkup, those who slept 5 h or less had higher odds of self-reported predisposition to the common cold compared to those who slept 7 h (14). More recently, a large longitudinal registry-based study found that a prior diagnosis of insomnia was associated with an increased risk of later being diagnosed with upper respiratory infections (17). This study included data from two population-based cohorts—the UK Biobank and FinnGen—with up to 23 years of follow-up data from primary care and/or hospitals. In the present study, we found an increased risk of self-reported antibiotic use for patients who reported sleep duration of < 6 h, chronic insomnia disorder or a chronic sleep problem. This may indicate that sleep disturbances are more strongly associated with more severe types of RTI (such as pneumonia). If our study had included more patients and if the included categories of RTI had been more specific, we might have detected effects of the sleep variables on the specific types of RTI.

We have not identified any previous studies that have specifically reported on the association between sleep parameters and the risk of gastroenteritis or UTI in an adult or patient population. Sleep disturbances are common in patients with gastrointestinal disease (30), and there is suggestive evidence from clinical trials that treatment of sleep disorders may improve gastrointestinal symptoms in patients with irritable bowel syndrome (31, 32). The questionnaire used in the present study asked the respondents whether they have had any “gastrointestinal infection with vomit or diarrhea”. This may be interpreted in different ways, but we think that most patients only reported acute gastrointestinal infections. Our results indicate an association between chronic insomnia disorder and the risk of UTI. Like for all infections explored in the present study, we were not able to assess the direction of this effect due to the cross-sectional design.

We found no clear associations between circadian preference and risk of infection or antibiotic use. We have not identified any previous studies on how circadian preference might affect the risk of infections. This question deserves further exploration.

### 4.3. Implications for research and practice

The results from the present study are in line with previous experimental studies in humans that have found increased risk of infection with sleep deprivation or insomnia. In two studies in which healthy adult individuals were infected with rhinovirus, those who had short sleep duration prior to the exposure of the virus were more likely to develop a clinical cold (8, 10). Similarly, previous studies have found a reduction in number of virus-specific antibody titers for influenza, hepatitis A, hepatitis B, and H1N1 (swine flu) in individuals with poor sleep before and after vaccination (33–36). Poor sleep can affect various immune parameters which in turn could reduce the body's ability to fight an infection (1, 18). A systematic review and meta-analysis of cohort and experimental studies on sleep disturbances, sleep duration and inflammation, reported an increase in the inflammatory markers C-reactive protein (CRP) and Interleukin 6 (IL-6) with the presence of sleep disturbance defined by use of validated questionnaires (18). The same study reported an association between long sleep duration (>8 h) and an increase in CRP and IL-6, while no evidence of increase in markers of inflammation were found for short sleep. There has been less focus on the effect of sleep disorders, such as insomnia, on immune response. Insomnia is very common among patients in general practice (5, 6), but under-recognized among GPs (37, 38). Cognitive behavioral therapy for insomnia (CBT-I) has been found to be highly effective in a primary care setting (39), and there is also suggestive evidence that such treatment can reduce the level of CRP in the blood (40). Recently, a more direct link between insomnia and risk of infection was demonstrated in a Mendelian randomization study with genetic data from a large Finnish cohort (17). By using genetic variants strongly associated with insomnia, the authors were able to assess the causal effect of insomnia on respiratory infections. They found that insomnia increased the risk of upper respiratory infection, and COVID-19 hospitalization and severity. Hence, interventions aimed at treating individuals with insomnia could potentially reduce the risk of infections.

Unlike in experimental studies, we were not able to exclude the role of potential unobserved confounding. An underlying health problem could affect both the risk of sleep problems and infections. For example, long sleep duration is associated with cardiovascular disease, diabetes and obesity (41), and has also been linked to depression, low education, low physical activity levels, and high drinking and smoking rates (42). Of these, we only had information on educational level. We view the results on chronic insomnia disorder as more robust than the results on sleep duration as insomnia is a long-term condition that is regarded as independent of other conditions (43). Although an underlying health problem could be a potential unobserved causal factor of both sleep problems and infection risk in the present study, it is likely that better sleep nevertheless could serve as a moderator reducing the risk of infection. More longitudinal studies in the general population and among patients in general practice, as well as clinical studies on the effect of treatment of insomnia on risk of infection, are needed. Data on different groups of infections and their potential differences in associations with sleep could give us important clues about potential underlying mechanisms.

## 5. Conclusions

In conclusion, we found a higher risk of infection in patients that reported short or long sleep duration, chronic insomnia, or a chronic sleep problem. The risk of antibiotic use was also increased in patients who reported short sleep duration, chronic insomnia disorder or a chronic sleep problem. Sleep could be a potential target when developing measures to prevent infections and reduce the use of antibiotics.

## Data availability statement

The raw data supporting the conclusions of this article will be made available by the authors, without undue reservation.

## Ethics statement

The studies involving human participants were reviewed and approved by the Regional Committee for Medical and Health Research Ethics in Western Norway (REK) (ref. 61165). The patients/participants provided their written informed consent to participate in this study.

## Author contributions

IF performed the data analysis and drafted the manuscript. All authors contributed to the interpretation of the data, reviewed, critically revised and approved the manuscript, take full responsibility for the work, the conduct of the study, controlled the decision to publish, conceived the idea for the present study, and contributed to the data collection.
