# Supplementary material for: The association between self-reported sleep problems, infection, and antibiotic use in patients in general practice
Source: Front Psychiatry. 2023 Mar 2;14:1033034. doi: 10.3389/fpsyt.2023.1033034 (PMC10017838; doi:10.3389/fpsyt.2023.1033034)
Supplement: Supplementary file 1 [file Table_1.docx]

**Supplemental material**

**Supplementary table 1:** Crude and adjusted relative risks (RR) with 95% confidence intervals (CI) for any type of infection and antibiotic 440 use among 1848 patients visiting their GPs in the spring and fall of 2020, statistically significant results are indicated in bold

|  | **Any type of infection** | | **Antibiotic use** | |
| --- | --- | --- | --- | --- |
| **Characteristics** | **Crude RR (95% CI)** | **Adjusted^a^ RR (95% CI)** | **Crude RR (95% CI)** | **Adjusted^a^ RR (95% CI)** |
| **Sleep duration** |  |  |  |  |
| <6 hours | **1.28 (1.13-1.45)** | **1.27 (1.11-1.46)** | **1.47 (1.10-1.98)** | **1.57 (1.13-2.18)** |
| 6-7 hours | 1.09 (0.98-1.23) | 1.10 (0.98-1.25) | 0.99 (0.75-1.30) | 1.04 (0.76-1.42) |
| 7-8 hours | 1.00 (ref.) | 1.00 (ref.) | 1.00 (ref.) | 1.00 (ref.) |
| 8-9 hours | 1.06 (0.86-1.31) | 1.12 (0.90-1.39) | 1.14 (0.71-1.84) | 1.12 (0.64-1.96) |
| >9 hours | **1.53 (1.23-1.90)** | **1.44 (1.12-1.84)** | 0.78 (0.30-2.02) | 0.94 (0.36-2.48) |
| **Chronic insomnia disorder** |  |  |  |  |
| Yes | **1.17 (1.07-1.28)** | **1.15 (1.05-1.27)** | **1.41 (1.13-1.75)** | **1.47 (1.16-1.87)** |
| No | 1.00 (ref.) | 1.00 (ref.) | 1.00 (ref.) | 1.00 (ref.) |
| **Chronic sleep problem** |  |  |  |  |
| Yes | **1.12 (1.02-1.22)** | **1.13 (1.03-1.24)** | **1.30 (1.05-1.62)** | **1.33 (1.05-1.69)** |
| No | 1.00 (ref.) | 1.00 (ref.) | 1.00 (ref.) | 1.00 (ref.) |
| **Circadian preference** |  |  |  |  |
| Morning type | 0.94 (0.84-1.06) | 0.97 (0.85-1.10) | 1.02 (0.78-1.35) | 0.93 (0.69-1.25) |
| Neither morning nor evening type | 1.00 (ref.) | 1.00 (ref.) | 1.00 (ref.) | 1.00 (ref.) |
| Evening type | 1.07 (0.95-1.20) | 1.05 (0.93-1.19) | 0.87 (0.66-1.17) | 0.90 (0.66-1.23) |
| ^a^Adjusted for sex, age and age squared, education, children living at home, season of data collection. | | | | |
